# Endoscopic Delivery Method Using a Retrieval Net for Patients with Small-Bowel Capsule Endoscopy Stagnation in the Stomach

**DOI:** 10.1155/2021/3216193

**Published:** 2021-12-16

**Authors:** Akihiko Sumioka, Shiro Oka, Akiyoshi Tsuboi, Issei Hirata, Sumio Iio, Hidenori Tanaka, Ken Yamashita, Takahiro Kotachi, Ryo Yuge, Yuji Urabe, Shinji Tanaka

**Affiliations:** ^1^Department of Gastroenterology and Metabolism, Hiroshima University Hospital, 1-2-3 Kasumi, Minami-ku, Hiroshima 734-8551, Japan; ^2^Division of Regeneration and Medicine Center for Translational and Clinical Research, Hiroshima University Hospital, 1-2-3 Kasumi, Minami-ku, Hiroshima 734-8551, Japan; ^3^Department of Endoscopy, Hiroshima University Hospital, 1-2-3 Kasumi, Minami-ku, Hiroshima 734-8551, Japan

## Abstract

With the increasing use of capsule endoscopy (CE), screening tests for the small bowel can be performed with minimal invasiveness. However, occasionally, the entire small bowel cannot be observed because of decreased peristalsis of the stomach. For such cases, we perform delivery of CE by an endoscope. We retrospectively examined the usefulness of the endoscopic delivery method using a retrieval net for patients with CE stagnation in the stomach. From 2,270 patients who underwent small-bowel CE at Hiroshima University Hospital from January 2013 to January 2020, 29 consecutive patients (1.3% of the total number) in whom the small bowel could not be observed due to CE stagnation in the stomach at the time of the initial CE underwent the endoscopic delivery method using a retrieval net for secondary small-bowel CE. This study included 16 male (55%) and 13 female (45%) patients with a mean age of 69.2 ± 13.2 years. 11 patients (38%) had a history of gastrointestinal surgical resection. The entire small bowel could be observed in 19 patients (66%), and CE reached the terminal ileum in the remaining patients. A history of gastrointestinal surgical resection was significantly more frequent in the group where the entire small bowel could not be observed. The rate of small-bowel lesion detection was 55% (16/29). There were no adverse events associated with our endoscopic delivery method. Thus, the endoscopic delivery method using a retrieval net for patients with initial CE stagnation in the stomach may be safe and useful for the detection of small-bowel lesions.

## 1. Introduction

Capsule endoscopy (CE) is convenient, minimally invasive, and free of radiation exposure, making it the first-line diagnostic tool for small-bowel observation [1–5]. The Japanese Gastroenterological Endoscopy Society (JGES) guidelines recommend CE as the first-line diagnostic tool for use in cases of obscure gastrointestinal bleeding (OGIB) [6]. Complete examination, whereby the CE traverses the entire small bowel and reaches the colon during the battery life, is achieved in 70%–80% of CE procedures [7, 8]. Because the CE is not self-propelled and moves through the gastrointestinal lumen with peristaltic movement, there are cases in which the entire small bowel cannot be observed because of stagnation in the stomach. CE stagnation in the stomach has not been frequently discussed in the literature, but a few reports suggest that the risk of this event is 0%–5% [9, 10]. In patients with CE stagnation in the stomach, prokinetics or endoscopic delivery method of the CE into the duodenum should be performed [11–13]. Double balloon endoscopy (DBE) is a relatively invasive method, and antegrade and retrograde procedures are sometimes required to observe the entire small bowel. We reported that CE and DBE exhibit a nearly equal ability to detect lesions if the entire small bowel is observed [5]. Lesions were detected in 68% patients who underwent CE and/or DBE, and 39% lesions were amenable to treatment; thus, observation of the entire small bowel is indispensable when small-bowel lesions are strongly suspected.

To our knowledge, there are few reports on the endoscopic delivery methods only for patients with CE stagnation in the stomach. At our hospital, the endoscopic delivery method to the duodenum is performed using a retrieval net in such patients. The purpose of this study was to clarify the effectiveness of our method in patients with CE stagnation in the stomach.

## 2. Materials and Methods

### 2.1. Patients

**Figure 1:**
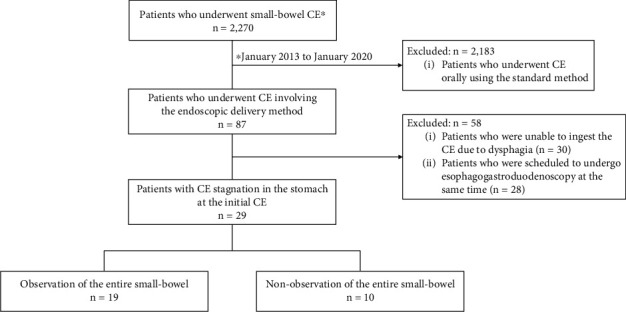
Flowchart of enrolled patients. CE: capsule endoscopy.

A flowchart of the enrolled patients is shown in Figure 1. A total of 2,270 patients who underwent small-bowel CE at Hiroshima University Hospital from January 2013 to January 2020 were included in this study. We excluded 2,183 patients that had undergone CE through standard oral ingestion. From the remaining 87 patients who underwent CE involving the endoscopic delivery method, we excluded 30 who could not ingest the CE due to dysphagia and 28 who were scheduled to undergo esophagogastroduodenoscopy at the same time. Thus, 29 consecutive patients (1.3% of the total number) in whom the small bowel could not be observed because of CE stagnation in the stomach at the initial CE were enrolled in this study.

This study was performed in accordance with the principles of the Declaration of Helsinki and approved by the Hiroshima University Hospital Institutional Review Board (registration number: E-2270). All patients were informed of the risks and benefits of CE, and each provided written informed consent for the procedure and the use of their deidentified data for research purposes.

### 2.2. Initial CE Procedure

Before the initial CE procedure, all patients underwent transabdominal ultrasonography and/or abdominal computed tomography (CT) to rule out small-bowel stenosis. CE was performed using a PillCam SB2 or SB3 video capsule (Covidien, Mansfield, MA, USA). The patient swallowed the CE with a solution of dimethicone after an overnight fast. Sodium picosulfate and magnesium citrate were administered for bowel preparation the night before the CE was swallowed. Only sodium picosulfate was prescribed to patients with renal dysfunction. The patients were instructed to swallow the CE in the sitting position, and they could resume their normal activities immediately thereafter. Images were analyzed using Rapid Reader 6.5 software running on a RAPID 8 workstation (Covidien, Mansfield, MA, USA). The CE was taken orally at 9:00, and the sensor arrays and recording device were removed at 17:00; this completed the examination. Total enteroscopy by CE was considered successful when the CE reached the cecum or the site of anastomosis in the ileocecal area within the recording time. In principle, we did not always check the position during the inspection using a real-time viewer (RTV). The capsule recordings were reviewed by two experienced physicians with an experience of reading more than 200 capsule videos. The diagnosis was established by consensus.

### 2.3. Secondary CE Involving the Endoscopic Delivery Method Using a Retrieval Net

**Figure 2:**
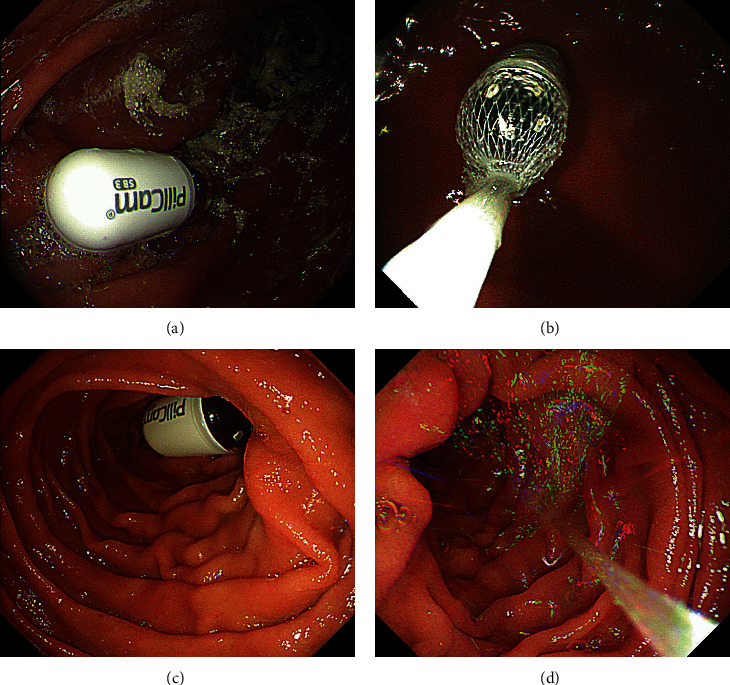
Procedure of the endoscopic delivery method using a retrieval net for small-bowel CE.

In the endoscopic delivery method, the CE was taken orally, following which an upper gastrointestinal endoscope (GIF-Q260J or GIF-H260; Olympus Corporation, Tokyo, Japan) was inserted. The CE was grasped at the stomach with a Roth Net (Olympus Medical Systems Corp, Japan), released in the descending part of the duodenum, and inserted to the depth of the transverse part of the duodenum with 200 ml of water (Figure 2). The presence of the CE in the small bowel was confirmed using RTV. None of the patients received antispasmodics. To minimize the influence of intestinal gas on CE images, carbon dioxide was used for endoscopic insufflation. The endoscopists who performed the procedure were not experienced in more than 100 esophagogastroduodenoscopies. As with the initial CE, the inspection started at 9:00 and ended at 17:00.

### 2.4. Evaluation

The clinical features of the enrolled patients, rate of entire small-bowel observation, rate of small-bowel lesion detection, lesions indicated for treatment, and adverse events related to the procedure were evaluated. CE stagnation in the stomach was considered present when the stomach was the last site reached during the initial examination with CE.

Data for each patient were obtained from a retrospective review of medical records. The final diagnosis of each patient was included in the respective medical chart. From the patients' medical records, data regarding the examinations and procedures, including CT, small-bowel follow-through, CE, and DBE, along with the operative specimen findings, were collected.

### 2.5. Statistical Analysis

Quantitative variables were compared using Pearson's chi-square test or Fisher's exact test. All tests were two-sided, and a *P* value of <0.05 was considered statistically significant. All statistical analyses were performed using JMP Pro 15 (SAS Institute Inc., Cary, NC, USA).

## 3. Results

Table 1 shows the characteristics of the enrolled patients. There were 16 male (55%) and 13 female (45%) patients with a mean age of 69.2 ± 13.2 years; these included 17 outpatients (59%) and 12 inpatients (41%). Among the inpatients, 9 patients (31%) and 3 patients (10%) underwent the procedure on the general medical floor and in the intensive care unit (ICU), respectively. Performance status (PS) was highest in 22 patients (75%) with PS0, and the body mass index (BMI) was highest in 16 patients (55%) with the normal range. The purpose of CE was OGIB in 15 patients (52%) and abdominal symptoms in 7 patients (24%). A history of gastrointestinal surgical resection was noted for 11 patients (38%). Comorbidities included cranial nerve disease in 13 patients (45%), heart disease in 8 patients (28%), diabetes in 7 patients (24%), collagen disease in 7 patients (24%), chronic kidney disease in 6 patients (21%), and hypothyroidism in 5 patients (17%).

The mean time from insertion of the upper gastrointestinal endoscope to insertion of the CE into the depth of the transverse part of the duodenum was 7.2 ± 3.4 min (Table 2). The entire small bowel was observed in 19 patients (66%), and CE reached the terminal ileum in the remaining patients. Therefore, it was considered possible to observe the entire small bowel using our endoscopic delivery method in almost all enrolled patients. The mean small-bowel transit time in complete CE was 337.4 ± 94.7 min. Overall, the rate of small-bowel lesion detection is 55% (16/29). The detected small-bowel lesions were as follows: angioectasia (6 patients, 21%), tumors (5 patients, 17%), inflammation (4 patients, 13%), and portal hypertensive enteropathy (1 patient, 4%). There were no adverse events associated with the endoscopic delivery method.

Among the 16 lesions detected by the endoscopic delivery method, angioectasia in 3 patients, diffuse large B cell lymphoma (DLBCL) in 1 patient, follicular lymphoma (FL) in 1 patient, and small-bowel polyps in 1 patient (Table 3) were indicated for treatment. All patients underwent endoscopic treatment or received a histopathological diagnosis using DBE. The 3 patients with angioectasia were treated with polidocanol injection and argon plasma coagulation without bleeding after treatment [14, 15]. DLBCL was treated with surgery and chemotherapy, and FL was treated with chemotherapy with achievement of complete remission. The small-bowel polyp was treated by endoscopic polypectomy, which resulted in curative resection.

We compared the entire small-bowel observation group with the nonobservation group in terms of the clinical characteristics of the enrolled patients (Table 4). A history of gastrointestinal surgical resection was significantly more frequent in the nonobservation group.

## 4. Discussion

The present study demonstrated that the endoscopic delivery method using a retrieval net was safe and useful for patients with CE stagnation in the stomach. Because CE propulsion depends on intestinal motility, the CE may not reach the cecum within the examination time, and the examination may be incomplete. Westerhof et al. reported risk factors for incomplete CE [16]. CE was incomplete in 19% (55/291) patients. The gastric transit time was significantly longer for patients with incomplete CE procedures than for those with complete CE procedures. A history of small-bowel surgery, hospitalization, moderate or poor bowel cleansing, and a gastric transit time of >45 min were identified as independent risk factors for incomplete CE procedures. Yazici et al. reported that inpatients have a significantly higher rate of incomplete CE than do outpatients, with a particularly high rate of incomplete CE among inpatients admitted to ICU [17]. This may be due to a number of factors, including generalized motor dysfunction, a sedentary status, and acute illnesses that affect hospitalized patients. They reported the gastric transit time and hospitalization as independent predictors of incomplete CE. Ben-Soussan et al. identified continuous hospitalization as a risk factor for gastric retention during CE [18]. Considering that delayed gastric transit has been recognized as a risk factor for incomplete small-bowel observation, we believe our endoscopic delivery method may improve the rate of entire small-bowel observation [16, 17].

Oral ingestion is the standard delivery method for CE, although the endoscopic delivery method for direct duodenal insertion has been developed for cases with abnormal gastric peristalsis, anatomical abnormalities, and dysphagia. Delivery devices such as the AdvanCE delivery device (US Endoscopy, Mentor, Ohio, USA) have been reported [19, 20]. Carey et al. reported the endoscopic delivery method using a Roth Net (US Endoscopy, Mentor, Ohio, USA) for 5 patients with gastroparesis, anatomical abnormalities, and dysphagia [21]. Although the endoscopic delivery to the duodenum was successfully completed in all 5 patients, the investigators encountered difficulties in releasing the CE from the retrieval net. In one instance, argon plasma coagulation was necessary to burn a hole in the net to release the CE. With our endoscopic delivery method using a retrieval net, the CE was easily released in the duodenum without any severe adverse events. Matsunaga et al. prospectively studied a CE delivery method using transnasal endoscopy without sedation [22]. The CE was delivered to the duodenum and released in 24 of 27 patients. In those 24 patients, the rate of entire small-bowel observation was significantly higher because of a shorter gastric transit time. Gao et al. reported that CE placement by conventional endoscopy improves the rate of entire small-bowel observation and the rate of small-bowel lesion detection [11]. When RTV confirmed that the CE was delayed in the esophagus or stomach for 1 h, the patients were sedated and a conventional endoscope was orally inserted to snare the CE and place it in the duodenum. Almeida et al. reported that the entire small-bowel observation rate with endoscopic placement was 77% (10/13) [23]. Gibbs and Bloomfeld studied 59 patients who underwent endoscopic placement and found that the rate of entire small-bowel observation was 64% (38/59) [12]. Among these cases, 21 involved initial CE stagnation in the stomach, and their rate of entire small-bowel observation was lower at 62% (13/21).

To our knowledge, there are few reports on endoscopic delivery methods only for patients with CE stagnation in the stomach. Even in previous reports, the rate of entire small-bowel observation with the endoscopic delivery methods for patients with CE stagnation in the stomach tended to be lower than that with the endoscopic delivery methods used for other causes [12]. In the endoscopic delivery method using a retrieval net in the present study, it was possible to observe almost the entire small bowel in patients with CE stagnation in the stomach. However, the rate of entire small-bowel observation in other patients during the same period was 78% (1702/2,183), tending to be lower in patients who underwent CE using endoscopic delivery method for CE stagnation in the stomach. The proportion (38%) of patients with a history of gastrointestinal surgical resection was higher in the present study than in previous reports [13, 24]; it is possible that the large proportion of patients with a history of gastrointestinal surgical resection resulted in a lower rate of entire small-bowel observation. Patients in whom the entire small bowel cannot be observed by the initial CE are often hospitalized or exhibit a decreased PS; thus, convenient and minimally invasive secondary CE is desirable, if possible. If the conditions for stagnation in the stomach can be predicted by analyzing a large number of cases, physicians can select the endoscopic delivery method for the initial CE itself.

This study has some limitations. First, this was a single-center, retrospective analysis. Second, the sample size was relatively small. Third, it was not possible to compare the results with those for cases of oral intake over the same period of time. Fourth, we did not use endoscopic delivery methods with the AdvanCE delivery device or polypectomy snares; therefore, we could not compare the usefulness of other delivery methods with that of our delivery method using a retrieval net. In addition, the amount of water and the type and amount of insufflation after CE release in the descending part of the duodenum should be considered.

## 5. Conclusions

The findings of this study suggest that the endoscopic delivery using a retrieval net is safe and useful for the detection of small-bowel lesions in patients with initial CE stagnation.

## Figures and Tables

**Table 1:** Characteristics of patients who underwent capsule endoscopy involving the endoscopic delivery method using a retrieval net.

Variables	Total (*n* = 29)
Sex	
Male	16 (55)
Female	13 (45)
Mean age ± SD (years)	69.2 ± 13.2
Inpatients	12 (41)
General medical floor	9 (31)
Intensive care unit	3 (10)
Performance status	
0	22 (75)
1	3 (10)
2	2 (7)
3	1 (4)
4	1 (4)
Body mass index	
Underweight	10 (35)
Normal range	16 (55)
Overweight	3 (10)
Purpose of CE	
Obscure gastrointestinal bleeding	15 (52)
Abdominal symptoms	7 (24)
Abnormalities on computed tomography	5 (17)
Other	2 (7)
History of gastrointestinal surgical resection	
Yes	11 (38)
No	18 (62)
Comorbidities (duplication)	
Cranial nerve disease	13 (45)
Heart disease	8 (28)
Diabetes	7 (24)
Collagen disease	7 (24)
Chronic kidney disease	6 (21)
Hypothyroidism	5 (17)
	(%)

**Table 2:** Mean time of the endoscopic delivery method, rate of entire small-bowel observation, mean small-bowel transit time in complete CE, detected small-bowel lesions, and adverse events in patients who underwent capsule endoscopy involving the endoscopic delivery method using a retrieval net.

Variables	Total (*n* = 29)
Mean time of endoscopic delivery method ± SD (min)	7.2 ± 3.4
Entire small-bowel observation	19 (66)
Mean small-bowel transit time in complete CE ± SD (min)	337.4 ± 94.7
Insertion to the terminal ileum	29 (100)
Detected small-bowel lesions	16 (55)
Angioectasia	6 (21)
Tumor	5 (17)
Small-bowel inflammation	4 (13)
Portal hypertensive enteropathy	1 (4)
Adverse events	0 (0)
	(%)

**Table 3:** Small-bowel lesions indicated for treatment after detection by capsule endoscopy involving the endoscopic delivery method using a retrieval net.

Case no.	Age (years)	Sex	Purpose of CE	Location	Detected lesion	Treatment method
1	62	Male	OGIB	Jejunum	Angioectasia	PDI+APC
2	62	Male	OGIB	Jejunum	Angioectasia	PDI+APC
3	68	Male	OGIB	Ileum	Angioectasia	PDI+APC
4	79	Female	Abdominal symptoms	Ileum	DLBCL	Surgery+chemotherapy
5	76	Male	Abdominal symptoms	Jejunum, ileum	Follicular lymphoma	Chemotherapy
6	37	Male	Abdominal symptoms	Jejunum, ileum	Polyp (hamartoma)	Polypectomy

OGIB: obscure gastrointestinal bleeding; PDI: polidocanol injection; APC: argon plasma coagulation; DLBCL: diffuse large B cell lymphoma.

**Table 4:** Comparisons between groups stratified by the observation or nonobservation of the entire small bowel by capsule endoscopy involving the endoscopic delivery method using a retrieval net.

Variables	Entire small-bowel observation		*P* value
	(Yes) (*n* = 19)	(No) (*n* = 10)	
Sex			
Male	12 (63)	4 (40)	0.270
Female	7 (37)	6 (60)	
Age (years)			
<65	5 (26)	3 (30)	1.000
≥65	14 (74)	7 (70)	
Inpatients	7 (37)	5 (50)	0.694
Performance status			
0–2	18 (95)	9 (90)	1.000
3–4	1 (5)	1 (10)	
Body mass index			
Underweight	6 (31)	4 (40)	0.901
Normal range	11 (58)	5 (50)	
Overweight	2 (11)	1 (10)	
Obscure gastrointestinal bleeding	10 (53)	5 (50)	1.000
History of gastrointestinal surgical resection			
Yes	4 (21)	7 (70)	0.017
No	15 (79)	3 (30)	
Cranial nerve disease			
Yes	8 (42)	5 (50)	0.714
No	11 (58)	5 (50)	
Diabetes			
Yes	5 (26)	2 (20)	1.000
No	14 (74)	8 (80)	
			(%)

## Data Availability

The data used to support the findings of this study are included within the article.
